# Biochemical Principles in Prion-Based Inheritance

**DOI:** 10.3390/epigenomes6010004

**Published:** 2022-01-25

**Authors:** Emily M. Dennis, David M. Garcia

**Affiliations:** 1 Department of Chemistry and Biochemistry, Institute of Molecular Biology, University of Oregon, Eugene, OR 97403, USA; edennis5@uoregon.edu; 2 Department of Biology, Institute of Molecular Biology, University of Oregon, Eugene, OR 97403, USA

**Keywords:** prion, epigenetics, transgenerational inheritance, prion-like, protein aggregation, amyloid

## Abstract

Prions are proteins that can stably fold into alternative structures that frequently alter their activities. They can self-template their alternate structures and are inherited across cell divisions and generations. While they have been studied for more than four decades, their enigmatic nature has limited their discovery. In the last decade, we have learned just how widespread they are in nature, the many beneficial phenotypes that they confer, while also learning more about their structures and modes of inheritance. Here, we provide a brief review of the biochemical principles of prion proteins, including their sequences, characteristics and structures, and what is known about how they self-template, citing examples from multiple organisms. Prion-based inheritance is the most understudied segment of epigenetics. Here, we lay a biochemical foundation and share a framework for how to define these molecules, as new examples are unearthed throughout nature.

## 1. Introduction

Prion proteins are a type of “protein-based inheritance” in which a conformational change in protein structure alters protein function and becomes self-templating without any requisite changes to DNA sequence. Prions are therefore an epigenetic phenomenon, albeit one not as well-studied as chromatin modifications, DNA chemical modifications, and certain classes of non-coding RNAs. Phenotypes that were later understood to be prion-based were studied as early as the 1960s [1], although it was not until the work of Stanley Prusiner and others that a more rigorous definition of their protein-only based nature was established. Prusiner’s work helped cement a protein expressed in the brain of mammals, known as PrP, as the defining example of a prion [2]. When this protein converts into a prionogenic conformation, however, it invariably leads to death, in the form of a variety of spongiform encephalopathies, including Creutzfeldt-Jakob Disease, Mad Cow Disease, Scrapie, and Chronic Wasting Disease. These diseases are highly infectious, and they can also occur spontaneously in very rare cases.

Today, our understanding of prion proteins is significantly more expansive—in rare cases they can cause disease, but there are even more examples of how they can be beneficial to organisms, permitting adaptation to environmental change [3], altered metabolism [4], increased cellular growth [5], and resistance to chemical stressors [6]. Prions also fundamentally alter our view of inheritance, as their ability to phenotypically alter an organism is based on heritable differences in protein structure and not any change to DNA sequence.

In this minireview, we focus our attention on the biochemical principles in prion-based inheritance. We outline sequence features of prions, their structures, and their aggregation/assembly properties. We hope this provides a useful introduction to non-experts, while supplying a framework for profiling features of newly discovered examples that we anticipate will further expand our understanding of this fascinating class of transgenerational molecules of inheritance.

## 2. Structural Motifs and Amino Acid Sequence Features Observed in Prion Proteins

A major shortcoming to understanding the general biochemical features of prion proteins is that only a handful of examples have been studied in detail. Nevertheless, some key features, sometimes predictive, are associated with prions and informative of the types of properties one could look for as new examples are discovered.

Prions can be grouped into two major classes based on the structural motifs that they can adopt: amyloid-forming prions and non-amyloid-forming prions [7]. Amyloid-forming prions are proteins that contain regions that can form highly ordered, β-sheet-rich protein aggregates [8,9,10,11]. Many proteins in nature can in fact form amyloids, many of them also with functional roles, although this feature does not make all of them prions that are capable of self-templating their protein conformation to other protein copies, which are in turn inherited across cell division or generations. Well-studied examples of amyloid-forming proteins in nature include Curli proteins in bacteria that are involved in biofilm formation [12]. Understanding amyloid structure or its mechanism of assembly into aggregates is underscored by the fact that dozens of human diseases are also associated with amyloid-forming proteins [13], including Aβ in Alzheimer’s disease [14], and α-synuclein in Parkinson’s disease [15]. It has been suggested that some of these amyloid-forming proteins may be bonafide prions, since they undergo a conformational change and self-assemble into structured protein aggregates that can spread across brain tissue, for example. These aggregates can even form proteinaceous seeds to propagate and contribute to the progression of disease [16]. It remains unclear, however, whether these mammalian amyloids meet other definitions of prions in the classical sense, such as being infectious to other organisms, as is the case with PrP in mammals, or for fungal prions. Indeed, this remains a fascinating proposition, while in our view, further investigation is needed to lend support to this prion hypothesis of neurodegenerative diseases, particularly with respect to their natural infectiousness from one organism or generation to another. From a molecular or evolutionary standpoint, the differences in how these proteins self-template their structures onto other copies, the reasons, and the outcomes, may indeed begin to blur with further study, perhaps encouraging reconsideration of what it means to be a “prion”.

The first prion protein identified, PrP^Sc^ (Sc = “scrapie”), forms amyloids and causes fatal brain spongiform encephalopathies in mammals [17]. After the discovery of PrP^Sc^, all prions discovered during approximately the next two decades were also found to form amyloid aggregates, in fungi, bacteria, and even viruses [18,19,20]. While the formation of amyloid structures universally reduced the activity of their encoded proteins, through sequestration of the protein into structured fibrils, the formation of these aggregates was not itself fatal to their host organisms, in contrast to the mammalian prion, PrP^Sc^. Moreover, altering the activity of these proteins often produced altered growth phenotypes that could be physiologically beneficial under a range of conditions [6,21,22]. More recently, through a different discovery pipeline, prion proteins lacking evidence of forming amyloids have been found in budding yeast [7]. When cells contain these proteins in their prion conformation, activity is often maintained, and sometimes is even increased [23,24]. The physical structures formed by these non-amyloid-forming prions are not well-understood, since only low-resolution methods have been used thus far. It also cannot be ruled out completely that these “non-amyloid” prions never form amyloid structures, even if only transiently. (A more extensive comparison of amyloid and non-amyloid prions is provided below.) A list of all known prions is provided in Table 1, separated by those that are amyloid-forming and those not known to form amyloids.

One common but not ubiquitous feature present in several prion protein sequences—including [URE3], [*PSI*^+^], [*RNQ*^+^]/[*PIN*^+^], [*MOT3*^+^], [*SWI*^+^], [*OCT*^+^] and Cb-Rho—is the enrichment of glutamine (Q) and/or asparagine (N). These Q/N-rich regions are most strongly associated with amyloid-forming prions in budding yeast, such that they have been predictive for identifying other potential prion candidates [3]. Alberti et al. performed a hybrid computational–experimental prion screen, in which Q/N-rich regions served as one principle selection criteria, and identified 24 proteins with prion-like qualities out of an initial candidate list of 200 genes computationally screened from the proteome. Five of these were already known to be prions from prior work, validating the approach while producing an additional 19 new prion candidates. While informative about the prevalence of these types of prion sequences across the proteome, one caveat of this approach was that the first discovered and perhaps most well-characterized prion protein, PrP, does not contain Q/N-rich regions, and therefore would not have emerged from such a screen. Other prions since identified—such as [*ISP*^+^], [*MOD*^+^], [*GAR*^+^], [*ESI*^+^], [*BIG*^+^] (Table 1), and others [7]—would also not have been “hits” in this screen, because their proteins also do not contain Q/N-rich regions.

Given their recent discovery, less is known about the sequence characteristics of non-amyloid-forming prions. Chakrabortee and colleagues reported in 2016 an experimental prion-discovery screen, in which there was no computational pre-filter for Q/N-rich sequences or other features typical of previously discovered fungal prions [7]. Instead, cells were screened for the capacity of nearly every protein in the proteome to individually elicit a long-lasting, epigenetic growth phenotype in response to transient overproduction of the protein. Among the dozens of “hits” emerging from this screen, the most consistent feature among the protein sequences were regions with high predicted disorder. Regions predicted to be structurally disordered, also known as “intrinsically disordered regions” (IDRs), do not resemble sequences found in structured portions of proteins. They are often depleted in hydrophobic amino acids that mediate co-operative folding. Additionally, they typically contain a higher proportion of polar or charged amino acids [35]. Notably these regions were frequently poorly conserved at the level of linear amino acid sequence, even while the IDR itself was maintained over vast evolutionary timescales.

For some prion proteins, specific regions, sometimes referred to as “prion domains” (PrDs), have been characterized. These regions can be both necessary and sufficient to impart a protein with prionogenic behavior, including when added exogenously to other proteins, even with GFP [3,36]. This modular nature—the regions are often separable from segments of the protein that carry out canonical activities—suggests the importance of structurally independent PrDs, and their role in prion conformational switching. Not all prions, however, contain such separable regions. For example, for the budding yeast protein Rnq1, which forms [*RNQ*^+^]/[*PIN*^+^], the regions involved in prion formation are not concentrated in one continuous segment of amino acid sequence, but instead are spread across multiple, distant segments in the protein [37]. Many PrDs are also predicted to be IDRs, and sometimes these two terms are used interchangeably. It is not conclusively known whether a high degree of structural disorder is a prerequisite for a PrD to be able to undergo conformational changes that endow it with prion behavior. It is also unlikely that all proteins that contain relatively large IDRs are prions. Disordered regions have been shown to play many other important roles, such as in the regulation of cell signaling [38,39], where these functions would not necessarily require them to be prions.

## 3. High-Resolution Structures of Prions or Prion-like Proteins

**Figure 1:**
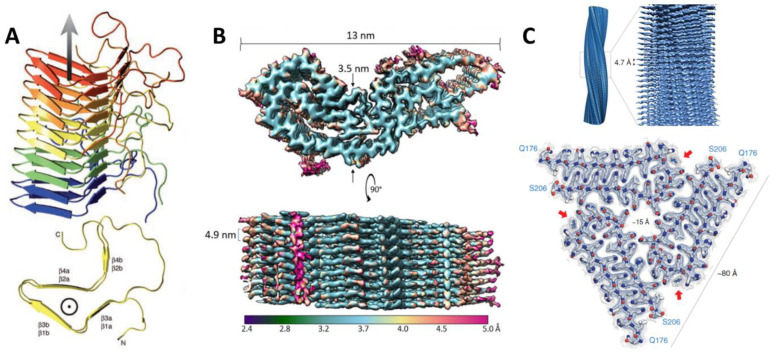
High-resolution structures of prion/prion-like amyloids. (**A**) [Het-s], reprinted from [42] with permission from AAAS. (**B**) PrP^Sc^, reprinted from [43] with permission from Elsevier. (**C**) Orb2, reprinted from [40] with permission from AAAS. As amyloids, these proteins show a similar packing of monomers along a fibril axis, however their core patterns vary considerably.

Few high-resolution structures of prions or prion-like proteins currently exist. Only three, all amyloid-forming, are known; one of these is a protein from fruit flies that has prion-like features [40] (Table 2). Their structural features vary significantly, and thus further investigations are needed before generalizable features of prion protein structure may be elucidated (Figure 1). The diversity of structural features may portend a larger number of potential prion proteins in proteomes. Investigating proteins with similar structural features to these is likely to be informative about the features that distinguish prions from non-prion proteins.

The first high-resolution structure of an infectious prion particle was the PrD of [Het-s], determined using solid state-NMR. [Het-s] is responsible for heterokaryon incompatibility in the filamentous fungus *Podospora anserina*. Its amyloid fibrils form a left-handed β-solenoid with a compact triangular hydrophobic core (residues 218–289) [41,42]. An unusual structural feature is the presence of pseudo-repeats of the β helix along the fibril axis, allowing a structure in which one molecule forms two turns of the solenoid. This early example of a functional amyloid was the first look into the structural features that govern prion amyloids.

The culprit of all known mammalian prion diseases, such as Creutzfeldt-Jakob Disease (CJD) in humans and Mad Cow Disease in bovines, PrP is perhaps the best characterized prion protein. A 3.1 Å structure of an infectious, hamster brain-derived PrP core protein was recently solved by Kraus and colleagues using cryo-electron microscopy, marking an important advancement in the study of how prion structure relates to its function [43]. PrP forms amyloid fibrils comprised of monomers with parallel, in-register, intermolecular β-sheets and connecting chains. This new structure differs from previous predictions from lower-resolution structures that PrP would contain β-solenoids or independent protofilaments, like other prion proteins [44,45,46,47,48]. In a point of particular interest, this high-resolution structure revealed more about the positioning of asparagine (N)-linked glycosylation and the C-terminal GPI anchor, indicating that they lie toward the lateral edges of the fibrils. The GPI anchor, which will be explored in more detail in the next section, localizes PrP to the membrane and glycosylation stabilizes the stacking of monomers to form amyloid fibrils, allowing for precise self-templating of monomers and faithful propagation of the prionogenic form of PrP. This high-resolution structure represents a huge leap in understanding of how the structure of the protein monomer may promote its self-templating function.

A high-resolution structure was also recently solved of the putative prion protein Orb2, a conserved RNA-binding protein, also known as CPEB in other animals, that plays a role in long-term potentiation/memory formation in *Drosophila melanogaster* [40,49]. The 2.6 Å cryo-EM structure shows that Orb2 forms 75 nm long, threefold-symmetric amyloid filaments. Of the two isoforms of Orb2 in the fly brain, Orb2A is less abundant, but seeds aggregation of the more abundant Orb2B. The protofilament core of Orb2B, which extends from residues 176 to 206, forms a hairpin fold made up of two β-strands with a wide turn between them. The conformational change induced by this structure converts the protein’s activity from a translational repressor into a translational activator. It was hypothesized that the hydrophilic, glutamine/histidine-rich fold could influence amyloid stability when the histidine residues are protonated, suggesting a mechanism for regulation. This structure provides a fascinating example of how an amyloid can serve as a stable, yet dynamic substrate of memory.

## 4. Conformational Switching, Self-Templating, and Factors Involved in Prion Propagation

Perhaps the most important step in prion formation is the conformational switching of a protein from its non-prion, or “naive” state, into a prionogenic, self-templating state. The human PrP protein has long been known to be able to switch from its native alpha conformation, characteristic of PrP^C^ (c = “cellular”), into a more compact, highly soluble, β-rich monomer, a precursor to larger multi-protein, insoluble fibrillar forms [50].

In the [*PSI*^+^] yeast prion, composed of the aggregates of the Sup35 translation termination factor, the intrinsically disordered and Q/N-rich “NM” domain at the N-terminus of the protein is necessary and sufficient for nucleation of prion “seeds”, or small multi-protein aggregates or assemblies that are capable of mediating transgenerational inheritance and growing into larger amyloid fibers. Assembly of these aggregates proceeds by monomer addition [51,52,53]. Within the NM-domain, also known as the PrD of Sup35, relatively short, specific sequences are required to nucleate the conversion of Sup35 from a soluble monomer into aggregates [54]. One recent study demonstrated how the size of the nucleation seed itself is also responsible for persistence of amyloid aggregation, highlighting how this biochemical feature influences transgenerational stability of the prion-based phenotypes [55].

While prions are commonly thought to self-template conformational changes in other copies of the same protein, there is also evidence for one prion protein seeding the formation of a second prion protein. In fact, formation of the [*PSI*^+^] prion in vivo requires pre-existing [*RNQ*^+^]/[*PIN*^+^], and there are particular sequences in both Sup35 and Rnq1 proteins that facilitate this cross-seeding [56]. [*RNQ*^+^]/[*PIN*^+^] can even promote amyloid formation of exogenously expressed proteins, such as those involved in some human neurological diseases [57].

There are also cases where cellular structures, such as membranes, are also key for promoting self-association of monomers that could increase the likelihood of conformational changes or self-templating behavior. PrP protein is anchored to the membrane via a GPI anchor in its C-terminus. Its conformational switching involves the peeling of the C-terminal β1-helix 1-β2 loop away from the helices 2 and 3, and the unspiraling of each of the helices to form the extended strands of the middle and disulfide β arches that are central to the structure of the prionogenic form, PrP^Sc^ [43]. PrP is not the only prion protein known to reside in membranes. [*GAR*^+^] is a prionogenic form of the highly abundant yeast plasma membrane protein Pma1, however it is not known to what extent its cellular localization influences its propensity for prion conversion or self-assembly [4].

Another important feature of prion propagation and inheritance, as established from many studies in budding yeast, is the requirement for protein chaperones. Amyloid-forming prions depend on the heat shock protein and disaggregase, Hsp104 [3,6,58], while non-amyloid-forming prions depend on Hsp70 and Hsp90 [4,7]. Thus, while prions are believed to undergo autonomous conformational switching and self-templating, their transgenerational inheritance depends on these important co-factors. For Hsp104 clients, the need arises for the amyloid aggregates to be divided sufficiently to facilitate passage during cell division [59,60,61]. For Hsp70 clients—i.e., non-amyloid-forming prions—the specific physical interactions that the protein chaperones have with prion proteins that enable their propagation are not known.

There is extensive evidence that amyloid-forming prion proteins can assume more than one conformational state in their self-templating form, known as the “strain” phenomenon. For example, the [*PSI*^+^] prion can undergo stable propagation of different strains, reflected in both the cellular phenotypes as well as their physical structures [22,37,62,63,64]. Similarly, the [URE3] prion has also been shown to contain different strains [62,65,66], as has [*RNQ*^+^]/[*PIN*^+^] [62]. The concept of prion strains is not well explored for the non-amyloid-forming prions (Table 1). This remains an important question for future studies, especially when coupled with structural studies of these more recently discovered prions.

Prion conformations are also reversible. While measurements of the frequencies of loss of the prion state are not widely known, some measurements suggest that they are relatively infrequent [67].

## 5. Contrasting Amyloid-Forming vs. Non-Amyloid Prions, and Methods to Identify Them

While high-resolution detail of prion protein structures from biophysical studies may be limited, there is evidence that they can form a variety of macromolecular structures in vivo. When taking into consideration proteins with prion-like domains, we can observe structures that vary from rigid amyloid fibrils, to gel-like condensates, to liquid–liquid assemblies [23,43,68,69]. As described above, prions can be generally categorized into amyloid-forming and non-amyloid-forming. Below, we discuss what is known about how these two classes of prions aggregate in vitro and in vivo and how they differ.

More is known about amyloid-forming prions, as they were amongst the first prions discovered [3,6,21,58,70]. Amyloid-forming prions can produce highly ordered β-sheets that lead to structured and thermodynamically stable fibrils [9,40,43]. As naïve monomeric protein is sequestered into filaments, this generally leads to loss of or inactivation of protein function. The amyloid structures formed are also resistant to certain physical and chemical perturbations, including temperature and detergents [71,72]. Indeed, an established property to test the formation of amyloids is testing a protein’s resistance to SDS detergent [6,23,71].

Less is known about non-amyloid-forming prions. They are sensitive to denaturants such as SDS, a property which can be used to distinguish them from amyloid-forming prions [3,7,23]. Genetic evidence also supports this distinct class of prions as not forming an amyloid intermediate that is essential for propagation of the prion structure. Curing of amyloid-forming prions can be accomplished through transient inhibition of the protein disaggregase chaperone, Hsp104. In contrast, the non-amyloid-forming prions can be cured by transient Hsp70 inhibition, but not Hsp104 inhibition [4,5,7]. IDRs are another feature of non-amyloid-forming prions that have been identified as drivers of prion phenotypes [7] (as noted earlier, these may also be found in amyloid-forming prions). For example, the IDR region in the [*SMAUG*^+^] prion drives its self-assembly properties, allowing gel-like condensates to form [23]. It is possible that the more dynamic structure of non-amyloid assemblies allows manipulation of phenotypic states without elimination of protein activity [5,7,23,24], permitting a regulatory paradigm of prion induction and loss in response to environmental cues. More characterization is needed before such conclusions can be established.

Biochemical techniques have been key to characterizing prion protein assemblies. Dyes that specifically bind to amyloids have been used to analyze the kinetic and structural properties of amyloid-forming prions. Compounds such as Thioflavin T (ThT) [73] and Congo Red (CR) [74] produce fluorescence when bound to amyloid β-sheets, making them an excellent method to detect the presence of amyloids. Another method to detect amyloid-forming prions is semi-denaturing detergent agarose gel electrophoresis (SDD-AGE) [75,76]. This technique exploits the fact that amyloid-forming prions are resistant to 2% SDS at room temperature, permitting visualization of the distribution of large biopolymer assemblies and monomeric protein. Additionally, it allows detection of amyloid fibers from cell lysate rather than from purified protein and has been used to characterize [*PSI*^+^], [*RNQ*^+^]/[*PIN*^+^], and [*MOT3*^+^] [77], among others.

Microscopy is another approach for characterizing prion aggregation, both in vivo and from purified protein [3,7]. GFP-fusion proteins allow visualization of prion aggregation in vivo, a useful tool for observing protein localization, aggregation formation, and induction of the prion state [78]. This has been used in characterizing [*PSI*^+^], [*MOT3*^+^], [*BIG*^+^], [*SMAUG*^+^] and more [3,5,23,79]. Fluorescent-reporter systems have also been developed for studying the kinetics of prion assembly formation, such as DAmFRET [80] and yTRAP [81], using [*PSI*^+^] to demonstrate their effectiveness. Several prions have been characterized using centrifugation, such as [*PSI*^+^] [79]. Combined with native separation techniques using sucrose gradients, the distribution of protein assemblies can be observed and quantified [82]. Altogether, these techniques have proven useful over the years to investigate the biochemical properties of prions.

## 6. Conclusions and Challenges

An inherent difficulty in the field of prion research is that, despite this phenomenon being described in the literature as early as the 1960s [1] and studied in fine detail in the case of PrP, insight into the basic biochemical properties has been somewhat shallow. Limitations in prion discovery have yielded too few examples from which to extract general principles. As prions cannot be unambiguously identified from DNA or protein sequences, from co-immunoprecipitations, from proteomics, from standard genetic screens, or from microscopy and other standard cell biological methods, new examples can be challenging to determine. We do not know of a discrete property of prions that could be used to identify them easily. Indeed, most descriptions of new examples involve a battery of tests that help exclude other possibilities that are consistent with the observed phenotypes, while also establishing known behavior associated with other prions.

Yet, we know that they are conserved across domains of life and several existing examples show that they exert a strong influence over biological processes. Without identifying and understanding more of them, we are overlooking a critical determinant of transgenerational inheritance and organismal function in nature.

In the early 1990s, when knowledge of the “histone code” and the general influence of chromatin modifications on the structure and function of genomes came to light, it was unlikely many could have foreseen the importance of this epigenetic code on both basic cellular functions and a myriad of human diseases. After decades of study, prions are a well-established biological phenomenon. Yet, the challenge of discovering them and measuring them remains, and we believe they must be explored more deeply to assess the extent of their broader impact in nature. Grounding our understanding of their inheritance and regulation in biochemical terms is an excellent starting point and should accelerate further discovery. It will be necessary to revisit these terms periodically, as new examples mature our understanding of this important but still underappreciated epigenetic phenomenon.

## Figures and Tables

**Table 1:** A summary of all known amyloid-forming prions and non-amyloid-forming prions. All are from budding yeast (*Saccharomyces cerevisiae*), except: ^1^ = mammals, ^2^ = *Podospora anserina*, ^3^ = *Clostridium botulinum*. Brackets denote the non-Mendelian inheritance of this element in genetic crosses, and capital letters denote its dominance.

Amyloid-Forming Prions	Non-Amyloid-Forming Prions
PrP^Sc^ [2] ^1^ [URE3] [25,26] [*PSI*^+^] [1,26] [*RNQ*^+^]/[*PIN*^+^] [27,28] [*MOT3*^+^] [3] [*MOD*^+^] [6] [Het-s] [29] ^2^ [*SWI*^+^] [30] [*OCT*^+^] [31] [*ISP*^+^] [32,33] [*NSI*^+^] [34] Cb-Rho [20] ^3^	[*GAR*^+^] [4] [*SMAUG*^+^] [23] [*ESI*^+^] [24] [*BIG*^+^] [5] Others [7]

**Table 2:** A summary of the features of the high-resolution prion/prion-like protein structures [40,42,43].

Prion/Protein	[Het-s]	PrP^Sc^	Orb2B
Gene name	*het* *-s*	*PRNP*	*orb2*
Core residues (aa)	218–289 (72)	95–227 (133)	176–206 (31)
Structure/Symmetry	Left-handed solenoid	Parallel in-register β-sheets	Threefold triangular symmetry
Method to interpret structure	ss-NMR	Cryo-EM	Cryo-EM
Core	Hydrophobic	Hydrophobic	Hydrophilic
Stabilizing features	23 hydrogen bonds, three salt bridges, two asparagine ladders	GPI anchor, “Greek Key” motif, β-arches	Interdigitated cross-β structure, protonation of histidine
Post-translational modifications	Unknown	*N*-linked glycosylation	Unknown

## References

[1] Cox B.S. (1965). Ψ, a Cytoplasmic Suppressor of Super-Suppressor in Yeast. Heredity.

[2] Prusiner S.B. (1982). Novel Proteinaceous Infectious Particles Cause Scrapie. Science.

[3] Alberti S., Halfmann R., King O., Kapila A., Lindquist S. (2009). A Systematic Survey Identifies Prions and Illuminates Sequence Features of Prionogenic Proteins. Cell.

[4] Brown J.C.S., Lindquist S. (2009). A Heritable Switch in Carbon Source Utilization Driven by an Unusual Yeast Prion. Genes Dev..

[5] Garcia D.M., Campbell E.A., Jakobson C.M., Tsuchiya M., Shaw E.A., DiNardo A.L., Kaeberlein M., Jarosz D.F. (2021). A Prion Accelerates Proliferation at the Expense of Lifespan. eLife.

[6] Suzuki G., Shimazu N., Tanaka M. (2012). A Yeast Prion, Mod5, Promotes Acquired Drug Resistance and Cell Survival Under Environmental Stress. Science.

[7] Chakrabortee S., Byers J.S., Jones S., Garcia D.M., Bhullar B., Chang A., She R., Lee L., Fremin B., Lindquist S. (2016). Intrinsically Disordered Proteins Drive Emergence and Inheritance of Biological Traits. Cell.

[8] Balbirnie M., Grothe R., Eisenberg D.S. (2001). An Amyloid-Forming Peptide from the Yeast Prion Sup35 Reveals a Dehydrated β-Sheet Structure for Amyloid. Proc. Natl. Acad. Sci. USA.

[9] Fitzpatrick A.W.P., Debelouchina G.T., Bayro M.J., Clare D.K., Caporini M.A., Bajaj V.S., Jaroniec C.P., Wang L., Ladizhansky V., Müller S.A. (2013). Atomic Structure and Hierarchical Assembly of a Cross-β Amyloid Fibril. Proc. Natl. Acad. Sci. USA.

[10] Glover J.R., Kowal A.S., Schirmer E.C., Patino M.M., Liu J.J., Lindquist S. (1997). Self-Seeded Fibers Formed by Sup35, the Protein Determinant of [PSI+], a Heritable Prion-like Factor of S. Cerevisiae. Cell.

[11] King C.-Y., Tittmann P., Gross H., Gebert R., Aebi M., Wüthrich K. (1997). Prion-Inducing Domain 2–114 of Yeast Sup35 Protein Transforms in Vitro into Amyloid-like Filaments. Proc. Natl. Acad. Sci. USA.

[12] Barnhart M.M., Chapman M.R. (2006). Curli Biogenesis and Function. Annu. Rev. Microbiol..

[13] Eisenberg D., Jucker M. (2012). The Amyloid State of Proteins in Human Diseases. Cell.

[14] Kayed R., Head E., Thompson J.L., McIntire T.M., Milton S.C., Cotman C.W., Glabe C.G. (2003). Common Structure of Soluble Amyloid Oligomers Implies Common Mechanism of Pathogenesis. Science.

[15] Steiner J.A., Quansah E., Brundin P. (2018). The Concept of Alpha-Synuclein as a Prion-like Protein: Ten Years After. Cell Tissue Res..

[16] Jucker M., Walker L.C. (2013). Self-Propagation of Pathogenic Protein Aggregates in Neurodegenerative Diseases. Nature.

[17] Kitamoto T., Tateishi J., Tashima T., Takeshita I., Barry R.A., DeArmond S.J., Prusiner S.B. (1986). Amyloid Plaques in Creutzfeldt-Jakob Disease Stain with Prion Protein Antibodies. Ann. Neurol..

[18] Garcia D.M., Jarosz D.F. (2014). Rebels with a Cause: Molecular Features and Physiological Consequences of Yeast Prions. FEMS Yeast Res..

[19] Nan H., Chen H., Tuite M.F., Xu X. (2019). A Viral Expression Factor Behaves as a Prion. Nat. Commun..

[20] Yuan A.H., Hochschild A. (2017). A Bacterial Global Regulator Forms a Prion. Science.

[21] Holmes D.L., Lancaster A.K., Lindquist S., Halfmann R. (2013). Heritable Remodeling of Yeast Multicellularity by an Environmentally Responsive Prion. Cell.

[22] True H.L., Lindquist S.L. (2000). A Yeast Prion Provides a Mechanism for Genetic Variation and Phenotypic Diversity. Nature.

[23] Chakravarty A.K., Smejkal T., Itakura A.K., Garcia D.M., Jarosz D.F. (2020). A Non-Amyloid Prion Particle That Activates a Heritable Gene Expression Program. Mol. Cell.

[24] Harvey Z.H., Chakravarty A.K., Futia R.A., Jarosz D.F. (2020). A Prion Epigenetic Switch Establishes an Active Chromatin State. Cell.

[25] Lacroute F. (1971). Non-Mendelian Mutation Allowing Ureidosuccinic Acid Uptake in Yeast. J. Bacteriol..

[26] Wickner R.B. (1994). [URE3] as an Altered URE2 Protein: Evidence for a Prion Analog in Saccharomyces Cerevisiae. Science.

[27] Derkatch I.L., Bradley M.E., Zhou P., Chernoff Y.O., Liebman S.W. (1997). Genetic and Environmental Factors Affecting the De Novo Appearance of the [Psi(+)] Prion in Saccharomyces Cerevisiae. Genetics.

[28] Derkatch I.L., Bradley M.E., Hong J.Y., Liebman S.W. (2001). Prions Affect the Appearance of Other Prions: The Story of [PIN+]. Cell.

[29] Coustou V., Deleu C., Saupe S., Begueret J. (1997). The Protein Product of the Het-s Heterokaryon Incompatibility Gene of the Fungus Podospora Anserina Behaves as a Prion Analog. Proc. Natl. Acad. Sci. USA.

[30] Du Z., Park K.-W., Yu H., Fan Q., Li L. (2008). Newly Identified Prion Linked to the Chromatin-Remodeling Factor Swi1 in Saccharomyces Cerevisiae. Nat. Genet..

[31] Patel B.K., Gavin-Smyth J., Liebman S.W. (2009). The Yeast Global Transcriptional Co-Repressor Protein Cyc8 Can Propagate as a Prion. Nat. Cell Biol..

[32] Rogoza T., Goginashvili A., Rodionova S., Ivanov M., Viktorovskaya O., Rubel A., Volkov K., Mironova L. (2010). Non-Mendelian Determinant [ISP+] in Yeast Is a Nuclear-Residing Prion Form of the Global Transcriptional Regulator Sfp1. Proc. Natl. Acad. Sci. USA.

[33] Volkov K.V., Aksenova A.Y., Soom M.J., Osipov K.V., Svitin A.V., Kurischko C., Shkundina I.S., Ter-Avanesyan M.D., Inge-Vechtomov S.G., Mironova L.N. (2002). Novel Non-Mendelian Determinant Involved in the Control of Translation Accuracy in *Saccharomyces cerevisiae*. Genetics.

[34] Saifitdinova A.F., Nizhnikov A.A., Lada A.G., Rubel A.A., Magomedova Z.M., Ignatova V.V., Inge-Vechtomov S.G., Galkin A.P. (2010). [NSI+]: A Novel Non-Mendelian Nonsense Suppressor Determinant in Saccharomyces Cerevisiae. Curr. Genet..

[35] Uversky V.N., Gillespie J.R., Fink A.L. (2000). Why Are? Natively Unfolded? Proteins Unstructured under Physiologic Conditions?. Proteins Struct. Funct. Genet..

[36] Li L., Lindquist S. (2000). Creating a Protein-Based Element of Inheritance. Science.

[37] Stein K.C., True H.L. (2014). Extensive Diversity of Prion Strains Is Defined by Differential Chaperone Interactions and Distinct Amyloidogenic Regions. PLoS Genet..

[38] Ba A.N.N., Yeh B.J., van Dyk D., Davidson A.R., Andrews B.J., Weiss E.L., Moses A.M. (2012). Proteome-Wide Discovery of Evolutionary Conserved Sequences in Disordered Regions. Sci. Signal..

[39] Oldfield C.J., Uversky V.N., Dunker A.K., Kurgan L., Salvi N. (2019). Chapter 1—Introduction to Intrinsically Disordered Proteins and Regions. Intrinsically Disordered Proteins.

[40] Hervas R., Rau M.J., Park Y., Zhang W., Murzin A.G., Fitzpatrick J.A.J., Scheres S.H.W., Si K. (2020). Cryo-EM Structure of a Neuronal Functional Amyloid Implicated in Memory Persistence in *Drosophila*. Science.

[41] Van Melckebeke H., Wasmer C., Lange A., Ab E., Loquet A., Böckmann A., Meier B.H. (2010). Atomic-Resolution Three-Dimensional Structure of HET-s(218-289) Amyloid Fibrils by Solid-State NMR Spectroscopy. J. Am. Chem. Soc..

[42] Wasmer C., Lange A., Van Melckebeke H., Siemer A.B., Riek R., Meier B.H. (2008). Amyloid Fibrils of the HET-s(218–289) Prion Form a β Solenoid with a Triangular Hydrophobic Core. Science.

[43] Kraus A., Hoyt F., Schwartz C.L., Hansen B., Artikis E., Hughson A.G., Raymond G.J., Race B., Baron G.S., Caughey B. (2021). High-Resolution Structure and Strain Comparison of Infectious Mammalian Prions. Mol. Cell.

[44] Amenitsch H., Benetti F., Ramos A., Legname G., Requena J.R. (2013). SAXS Structural Study of PrP(Sc) Reveals ~11 Nm Diameter of Basic Double Intertwined Fibers. Prion.

[45] Sim V.L., Caughey B. (2009). Ultrastructures and Strain Comparison of Under-Glycosylated Scrapie Prion Fibrils. Neurobiol. Aging.

[46] Spagnolli G., Rigoli M., Orioli S., Sevillano A.M., Faccioli P., Wille H., Biasini E., Requena J.R. (2019). Full Atomistic Model of Prion Structure and Conversion. PLoS Pathog..

[47] Terry C., Harniman R.L., Sells J., Wenborn A., Joiner S., Saibil H.R., Miles M.J., Collinge J., Wadsworth J.D.F. (2019). Structural Features Distinguishing Infectious Ex Vivo Mammalian Prions from Non-Infectious Fibrillar Assemblies Generated In Vitro. Sci. Rep..

[48] Vázquez-Fernández E., Vos M.R., Afanasyev P., Cebey L., Sevillano A.M., Vidal E., Rosa I., Renault L., Ramos A., Peters P.J. (2016). The Structural Architecture of an Infectious Mammalian Prion Using Electron Cryomicroscopy. PLoS Pathog..

[49] Si K., Kandel E.R. (2016). The Role of Functional Prion-Like Proteins in the Persistence of Memory. Cold Spring Harb. Perspect. Biol..

[50] Jackson G.S., Hosszu L.L.P., Power A., Hill A.F., Kenney J., Saibil H., Craven C.J., Waltho J.P., Clarke A.R., Collinge J. (1999). Reversible Conversion of Monomeric Human Prion Protein Between Native and Fibrilogenic Conformations. Science.

[51] Bradley M.E., Liebman S.W. (2004). The Sup35 Domains Required for Maintenance of Weak, Strong or Undifferentiated Yeast [PSI+] Prions. Mol. Microbiol..

[52] Collins S.R., Douglass A., Vale R.D., Weissman J.S. (2004). Mechanism of Prion Propagation: Amyloid Growth Occurs by Monomer Addition. PLoS Biol..

[53] Krishnan R., Lindquist S.L. (2005). Structural Insights into a Yeast Prion Illuminate Nucleation and Strain Diversity. Nature.

[54] Tessier P.M., Lindquist S. (2007). Prion Recognition Elements Govern Nucleation, Strain Specificity and Species Barriers. Nature.

[55] Villali J., Dark J., Brechtel T.M., Pei F., Sindi S.S., Serio T.R. (2020). Nucleation Seed Size Determines Amyloid Clearance and Establishes a Barrier to Prion Appearance in Yeast. Nat. Struct. Mol. Biol..

[56] Keefer K.M., Stein K.C., True H.L. (2017). Heterologous Prion-Forming Proteins Interact to Cross-Seed Aggregation in Saccharomyces Cerevisiae. Sci. Rep..

[57] Serio T.R. (2018). [PIN+]Ing down the Mechanism of Prion Appearance. FEMS Yeast Res..

[58] Chernoff Y.O., Lindquist S.L., Ono B., Inge-Vechtomov S.G., Liebman S.W. (1995). Role of the Chaperone Protein Hsp104 in Propagation of the Yeast Prion-Like Factor [ *Psi*+]. Science.

[59] Shorter J., Lindquist S. (2004). Hsp104 Catalyzes Formation and Elimination of Self-Replicating Sup35 Prion Conformers. Science.

[60] Shorter J., Lindquist S. (2006). Destruction or Potentiation of Different Prions Catalyzed by Similar Hsp104 Remodeling Activities. Mol. Cell.

[61] Shorter J., Lindquist S. (2008). Hsp104, Hsp70 and Hsp40 Interplay Regulates Formation, Growth and Elimination of Sup35 Prions. EMBO J..

[62] Bradley M.E., Edskes H.K., Hong J.Y., Wickner R.B., Liebman S.W. (2002). Interactions among Prions and Prion “Strains” in Yeast. Proc. Natl. Acad. Sci. USA.

[63] Derkatch I.L., Chernoff Y.O., Kushnirov V.V., Inge-Vechtomov S.G., Liebman S.W. (1996). Genesis and Variability of [Ps1] Prion Factors in Saccharomyces Cerevisiae. Genetics.

[64] Tanaka M., Chien P., Naber N., Cooke R., Weissman J.S. (2004). Conformational Variations in an Infectious Protein Determine Prion Strain Differences. Nature.

[65] Schlumpberger M., Prusiner S.B., Herskowitz I. (2001). Induction of Distinct [URE3] Yeast Prion Strains. Mol. Cell. Biol..

[66] Wickner R.B., Son M., Edskes H.K. (2019). Prion Variants of Yeast Are Numerous, Mutable, and Segregate on Growth, Affecting Prion Pathogenesis, Transmission Barriers, and Sensitivity to Anti-Prion Systems. Viruses.

[67] Derdowski A., Sindi S.S., Klaips C.L., DiSalvo S., Serio T.R. (2010). A Size Threshold Limits Prion Transmission and Establishes Phenotypic Diversity. Science.

[68] Alberti S. (2017). Phase Separation in Biology. Curr. Biol..

[69] Franzmann T.M., Jahnel M., Pozniakovsky A., Mahamid J., Holehouse A.S., Nüske E., Richter D., Baumeister W., Grill S.W., Pappu R.V. (2018). Phase Separation of a Yeast Prion Protein Promotes Cellular Fitness. Science.

[70] Toyama B.H., Kelly M.J.S., Gross J.D., Weissman J.S. (2007). The Structural Basis of Yeast Prion Strain Variants. Nature.

[71] Kryndushkin D.S., Alexandrov I.M., Ter-Avanesyan M.D., Kushnirov V.V. (2003). Yeast [PSI+] Prion Aggregates Are Formed by Small Sup35 Polymers Fragmented by Hsp104. J. Biol. Chem..

[72] Kushnirov V.V., Dergalev A.A., Alexandrov A.I. (2020). Proteinase K Resistant Cores of Prions and Amyloids. Prion.

[73] Xue C., Lin T.Y., Chang D., Guo Z. (2017). Thioflavin T as an Amyloid Dye: Fibril Quantification, Optimal Concentration and Effect on Aggregation. R. Soc. Open Sci..

[74] Yakupova E.I., Bobyleva L.G., Vikhlyantsev I.M., Bobylev A.G. (2019). Congo Red and Amyloids: History and Relationship. Biosci. Rep..

[75] Halfmann R., Lindquist S. (2008). Screening for Amyloid Aggregation by Semi-Denaturing Detergent-Agarose Gel Electrophoresis. J. Vis. Exp..

[76] Hanna-Addams S., Wang Z. (2018). Use of Two Dimensional Semi-Denaturing Detergent Agarose Gel Electrophoresis to Confirm Size Heterogeneity of Amyloid or Amyloid-like Fibers. J. Vis. Exp. JoVE.

[77] Halfmann R., Jarosz D.F., Jones S.K., Chang A., Lancaster A.K., Lindquist S. (2012). Prions Are a Common Mechanism for Phenotypic Inheritance in Wild Yeasts. Nature.

[78] Greene L., Park Y.-N., Masison D., Eisenberg E. (2009). Application of GFP-Labeling to Study Prions in Yeast. Protein Pept. Lett..

[79] Patino M.M., Liu J.-J., Glover J.R., Lindquist S. (1996). Support for the Prion Hypothesis for Inheritance of a Phenotypic Trait in Yeast. Science.

[80] Khan T., Kandola T.S., Wu J., Venkatesan S., Ketter E., Lange J.J., Rodríguez Gama A., Box A., Unruh J.R., Cook M. (2018). Quantifying Nucleation In Vivo Reveals the Physical Basis of Prion-like Phase Behavior. Mol. Cell.

[81] Newby G.A., Kiriakov S., Hallacli E., Kayatekin C., Tsvetkov P., Mancuso C.P., Bonner J.M., Hesse W.R., Chakrabortee S., Manogaran A.L. (2017). A Genetic Tool to Track Protein Aggregates and Control Prion Inheritance. Cell.

[82] Liebman S.W., Bagriantsev S.N., Derkatch I.L. (2006). Biochemical and Genetic Methods for Characterization of [PIN+] Prions in Yeast. Methods.

